# Supplementary material for: Prediction of recurrent stroke among ischemic stroke patients with atrial fibrillation: Development and validation of a risk score model
Source: PLoS One. 2021 Oct 8;16(10):e0258377. doi: 10.1371/journal.pone.0258377 (PMC8500448; doi:10.1371/journal.pone.0258377)
Supplement: S1 Fig — (PDF) [file pone.0258377.s001.pdf]

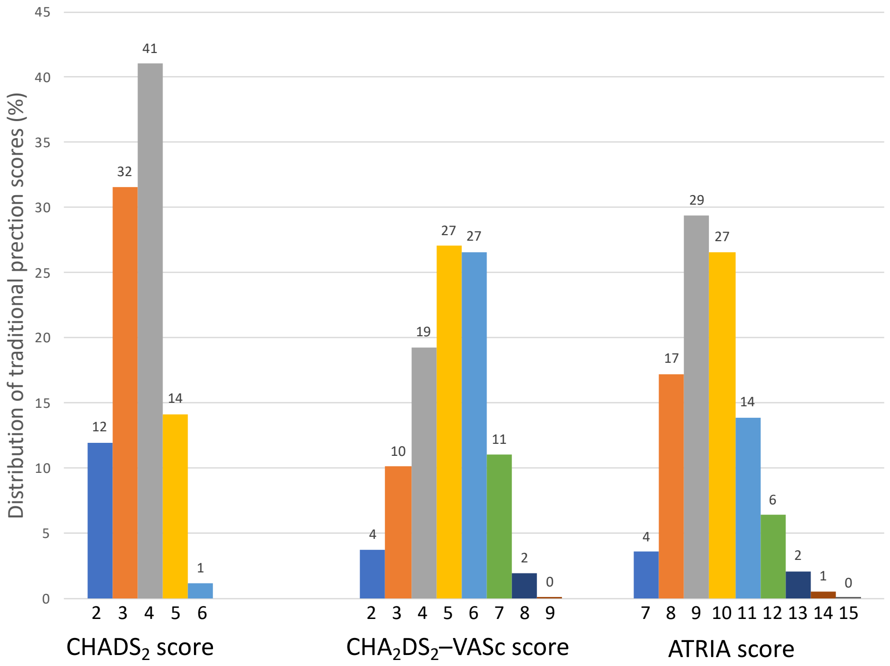

S1 Fig. Distribution of CHADS2, CHA2DS2-VASc, and ATRIA scores in the developmental dataset
