# Supplementary material for: Prediction of recurrent stroke among ischemic stroke patients with atrial fibrillation: Development and validation of a risk score model
Source: PLoS One. 2021 Oct 8;16(10):e0258377. doi: 10.1371/journal.pone.0258377 (PMC8500448; doi:10.1371/journal.pone.0258377)
Supplement: S2 Fig — (PDF) [file pone.0258377.s002.pdf]

$CHADS_2$ score

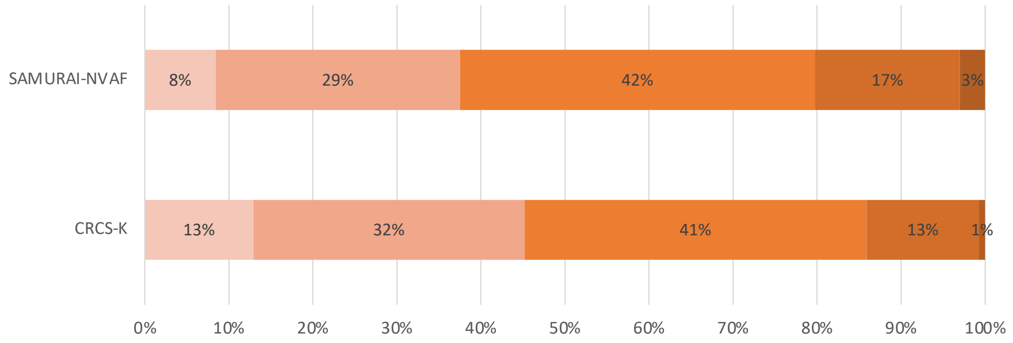

	CRCS-K	SAMURAI-NVAF
2	13%	8%
3	32%	29%
4	41%	42%
5	13%	17%
6	1%	3%

CHA_2DS_2-VASc score

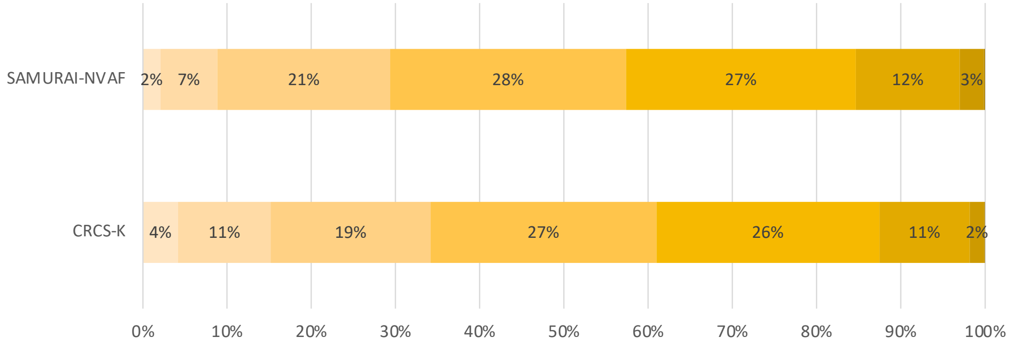

	CRCS-K	SAMURAI-NVAF
2	4%	2%
3	11%	7%
4	19%	21%
5	27%	28%
6	26%	27%
7	11%	12%
8	2%	3%
9	0%	0%

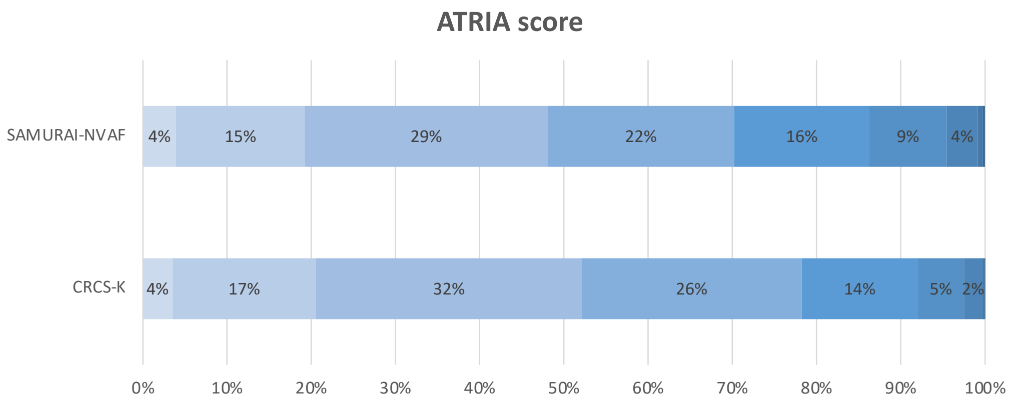

	CRCS-K	SAMURAI-NVAF
7	4%	4%
8	17%	15%
9	32%	29%
10	26%	22%
11	14%	16%
12	5%	9%
13	2%	4%
14	0%	1%
15	0%	0%

S2 Fig. Distribution of CHADS2, CHA2DS2-VASc, and ATRIA scores in the developmental dataset by countries
