# Supplementary material for: Prediction of recurrent stroke among ischemic stroke patients with atrial fibrillation: Development and validation of a risk score model
Source: PLoS One. 2021 Oct 8;16(10):e0258377. doi: 10.1371/journal.pone.0258377 (PMC8500448; doi:10.1371/journal.pone.0258377)
Supplement: S1 Table — (PDF) [file pone.0258377.s003.pdf]

S1 Table. Clinical profile of the external validation dataset

	External validation set (CRCS-K, 2015 - 2018) (N, 3668)
age	74.0 ± 10.2
male	1922 (52.4%)
Onset to arrival (d)	
≤24 hours	2916 (79.5%)
1 - 2 day	521 (14.2%)
≥3 days	231 (6.3%)
BMI	23.5 ± 3.5
TIA as an index stroke	88 (2.4%)
Pre-stroke mRS≥1	856 (23.3%)
NIHSS score	7 [2 - 15]
Hypertension	2609 (71.1%)
Diabetes	1112 (30.3%)
dyslipidemia	1005 (27.4%)
Smoking	954 (26.0%)
Newly detected Af	1710 (46.6%)
pre-stroke antiplatelets	1203 (32.8%)
pre-stroke anticoagulation	818 (22.3%)
Arterial occlusions	
ICA or MCA	1792 (48.9%)
Vertebro-basilar arteries	287 (7.8%)
Others	339 (9.2%)
Discharge medications	
aspirin	832 (22.7%)
clopidogrel	320 (8.7%)
cilostazol	105 (2.9%)
warfarin	780 (23.1%)
apixaban	718 (21.2%)
dabigatran	560 (16.6%)
rivaroxaban	511 (15.1%)
Laboratory information	
White blood cell count	8219 ± 2991
hemoglobin	13.4 ± 2.1
Total cholesterol	158 ± 38
Creatinine	1.08 ± 1.01
Initial glucose at arrival	142 ± 26
Systolic blood pressure	143 ± 26

Diastolic blood pressure	83 ± 16
$CHADS_2$ score	4 [3 - 4]
CHA_2DS_2-VASc score	5 [4 - 6]
ATRIA score	9 [9 - 10]
mRS score at discharge	
0	438 (11.9%)
1	652 (17.8%)
2	620 (16.9%)
3	481 (13.1%)
4	719 (19.6%)
5	758 (20.7%)
Recurrent stroke	157 (4.3%)
F/U duration for stroke	362 [161 - 365]
Death up to 1 year	400 (10.9%)
F/U duration for death (day)	364 [259 - 365]
