# Supplementary material for: Prediction of recurrent stroke among ischemic stroke patients with atrial fibrillation: Development and validation of a risk score model
Source: PLoS One. 2021 Oct 8;16(10):e0258377. doi: 10.1371/journal.pone.0258377 (PMC8500448; doi:10.1371/journal.pone.0258377)
Supplement: S1 File — The model’s predictability was compared to the observed probability of recurrent stroke stratified by the deciles of predicted risks. (PDF) [file pone.0258377.s004.pdf]

S1 File. Development, recalibration, and revision processes of the prediction model. The model's predictability was compared to the observed probability of recurrent stroke stratified by the deciles of predicted risks.

Table 1. The difference of observed and predicted probability in the developmental dataset (n, 5648)

Deciles of predicted risk	Observed event	Observed probability in developmental set (%)	Predicted probability for the developmental set (%)	Difference
1	10	1.77	2.06	-0.29
2	15	2.66	3.20	-0.54
3	23	4.07	3.88	0.19
4	24	4.25	4.50	-0.25
5	24	4.25	5.14	-0.89
6	26	4.60	5.89	-1.29
7	47	8.32	6.62	1.70
8	46	8.14	7.83	0.31
9	43	7.61	9.71	-2.09
10	80	14.16	16.20	-2.04
Total	338			

Table 2. The difference of observed and predicted probability in the external validation dataset (n, 3668)

Deciles of predicted risk	Observed event	Observed probability in the external validation set (%)	Predicted probability for external validation set (%)	Difference
1	7	1.91	1.89	0.02
2	9	2.45	3.14	-0.69
3	14	3.82	3.92	-0.11
4	18	4.91	4.54	0.37
5	16	4.36	5.13	-0.77
6	17	4.63	5.76	-1.13
7	17	4.63	6.92	-2.28
8	17	4.63	7.99	-3.36
9	18	4.91	10.15	-5.24
10	24	6.54	16.59	-10.05
Total	157			

Table 3. The differences of observed and predicted probability in the external validation dataset after calibrating model's overall slope

Deciles of predicted risk	Observed event	Observed probability in the external validation set (%)	Predicted probability for external validation set (%)	Difference
1	7	1.91	0.55	1.36
2	9	2.45	0.91	1.54
3	14	3.82	1.14	2.68
4	18	4.91	1.32	3.59
5	16	4.36	1.49	2.87
6	17	4.63	1.67	2.96
7	17	4.63	2.01	2.63
8	17	4.63	2.32	2.32
9	18	4.91	2.94	1.96
10	24	6.54	4.81	1.73
Total	157			

Table 4. The difference of observed and predicted probability in the external validation dataset after revising regression coefficients of the calibrated model

Deciles of predicted risk	Observed event	Observed probability in the external validation set (%)	Predicted probability for external validation set (%)	Difference
1	4	1.09	4.85	-3.76
2	9	2.45	6.49	-4.04
3	10	2.73	4.22	-1.50
4	5	1.36	6.30	-4.93
5	8	2.18	5.94	-3.76
6	14	3.82	6.41	-2.60
7	20	5.45	5.48	-0.03
8	23	6.27	6.74	-0.47
9	28	7.63	8.40	-0.77
10	36	9.81	11.22	-1.41
Total	157			
